# Acute Pleuro-Pneumonia, Producing Empyemia and Consolidation of the Lungs

**Published:** 1865-08

**Authors:** E. R. Travers

**Affiliations:** Amboy, Ill.


### ACUTE PLEURO-PNEUMONIA,

### PRODUCING EMPYEMIA AND CONSOLIDATION OF THE LUNGS.

By E. R. TRAVERS, M. D., Amboy, Ill.

March 21st, 1864, I was called to examine a patient, Mr. Harrison Pratt, aged 22, who had been sick about six weeks, and treated by Homœopathic medical skill during this period, when he was given up to die by that fraternity. I found him laboring with hurried and difficult breathing, a low, fluttering pulse, and suffering with acute pain. The left side of his chest was abnormally enlarged, while the heart occupied the right side; respiratory movement was confined to the right side; the left side was dull on percussion, from the scapular to the lower edge of the false ribs. I discovered fluctuation between the fourth and fifth ribs on the left side, and made it known to him and his friends. I then carefully introduced a grooved needle in a direction obliquely upwards and inwards, just below the angle of the fourth rib, when pus flowed out freely, of a thick, white appearance and fœtid odor. I then introduced the trochar in the same place, and drew off *six quarts of matter*, and injected the cavity with a solution of comp. tinc. of Iodine and rain water, in the proportion of one ounce of the tincture to a pint of water, and painted the surface affected with the comp. tinc. Iodine. I ordered him to take ten drops of the syrup of the Iodide of Iron three times a day, with Cod Liver Oil freely, beef tea, wine and oat-meal porridge.

In two weeks afterwards I again introduced the trochar and drew off *two gallons* of the same fœtid matter, of a somewhat thinner consistence; and in one week later I repeated the operation, and drew off *six quarts* more, which made *five gallons* in all, besides a large quantity that oozed out after each operation for several days. I continued to paint the surface with the comp. tinc. Iodine, also injecting the cavity with the solution above mentioned.

My patient was enabled to walk about the city and ride in a buggy, after the second operation. He expressed himself very thankful and happy after each evacuation of pus. He has continued to improve in health and strength ever since, and has removed to his father's home in the State of Pennsylvania, where he is now enjoying tolerably good health.

